# Poorly Differentiated Medullary Phenotype Predicts Poor Survival in Early Lymph Node-Negative Gastro-Esophageal Adenocarcinomas

**DOI:** 10.1371/journal.pone.0168237

**Published:** 2016-12-28

**Authors:** Christoph Treese, Pedro Sanchez, Patricia Grabowski, Erika Berg, Hendrik Bläker, Martin Kruschewski, Oliver Haase, Michael Hummel, Severin Daum

**Affiliations:** 1 Department of Gastroenterology, Infectious Diseases and Rheumatology, Charité University Medicine Berlin, Campus Benjamin Franklin, Berlin, Germany; 2 Berlin Institute of Health (BIH), Berlin, Germany; 3 Department of Gastroenterology and Endocrinology, Zentralklinik Bad Berka GmbH, Bad Berka, Germany; 4 Institute for Pathology, Charité University Medicine Berlin, Campus Benjamin Franklin, Berlin, Germany; 5 Department of General, Visceral and Thoracic Surgery, Städtisches Klinikum Solingen GmbH, Solingen, Germany; 6 Department of General, Visceral, Vascular and Thoracic Surgery, Charité University Medicine Berlin, Campus Mitte, Berlin, Germany; Shanghai Jiao Tong University School of Medicine, CHINA

## Abstract

**Background:**

5-year survival rate in patients with early adenocarcinoma of the gastro-esophageal junction or stomach (AGE/S) in Caucasian patients is reported to be 60–80%. We aimed to identify prognostic markers for patients with UICC-I without lymph-node involvement (N0).

**Methods:**

Clinical data and tissue specimen from patients with AGE/S stage UICC-I-N0, treated by surgery only, were collected retrospectively. Tumor size, lymphatic vessel or vein invasion, grading, classification systems (WHO, Lauren, Ming), expression of BAX, BCL-2, CDX2, Cyclin E, E-cadherin, Ki-67, TP53, TP21, SHH, Survivin, HIF1A, TROP2 and mismatch repair deficiency were analyzed using tissue microarrays and correlated with overall and tumor related survival.

**Results:**

129 patients (48 female) with a mean follow-up of 129.1 months were identified. 5-year overall survival was 83.9%, 5-year tumor related survival was 95.1%.

Poorly differentiated medullary cancer subtypes (p<0.001) and positive vein invasion (p<0.001) were identified as risk factors for decreased overall—and tumor related survival. Ki-67 (p = 0.012) and TP53 mutation (p = 0.044) were the only immunohistochemical markers associated with worse overall survival but did not reach significance for decreased tumor related survival.

**Conclusion:**

In the presented study patients with AGE/S in stage UICC-I-N0 had a better prognosis as previously reported for Caucasian patients. Poorly differentiated medullary subtype was associated with reduced survival and should be considered when studying prognosis in these patients.

## Introduction

Adenocarcinoma of the esophago-gastric junction and stomach (AEG/S) is the fourth most common cancer worldwide in men and the fifth most common cancer diagnosis in women [1]. The prognosis of patients with AEG/S is poor, making it the second leading cause of cancer death worldwide [1].

Complete resection (R0) is considered the treatment of choice with curative intent, yet, in up to 40% of patients tumor recurrence occurs even after R0 resection [2]. An extended D2 lymphadenectomy as surgical procedure is well established, confirming the role of lymphatic invasion in this cancer entity [3,4]. Approximately 30% of patients with AEG/S exhibit stage UICC I at time of diagnosis [5] and despite early tumor-stage and radical tumor resection, this group of patients has a 5-year overall survival rate of only 60%–80% [6,7]. In these studies lymph node involvement was the main risk factor for tumor relapse. Due to higher incidence of this cancer type in East Asia, most studies on prognosis and survival describe patients from Japan and China. Solely Borie et al. [8] studied the survival of Caucasian patients stage UICC I stratified according to lymph node involvement and demonstrated a tumor related five year survival of 92% in patients without lymph node involvement.

In the search for additional and refined prognostic risk factors in advanced tumor stages, several studies analyzed histomorphological criteria like tumor size, lymphatic vessel invasion, vein invasion, grading, Lauren classification, WHO classification, and MING classification [9–14]. Several studies described molecular markers considered predictive for survival of which we selected for immunohistochemical evaluation and categorized in subgroups: markers for proliferation (Ki-67 [15]), cell cycle regulation (Cyclin E [16–18], p21 [19], TP53 [20]), apoptosis (BAX [21], BCL [22], Survivin [23–25]), cell adhesion (E-cadherin [26–28]), cell differentiation (CDX2) [29], SHH (Sonic hedgehog) [30], TROP2 [31], HIF1A (hypoxia induced factor 1α) [32] and mismatch repair deficiency [33].

These biomarkers have not yet been analyzed in this subgroup with AEG/S, UICC I without the main confounder lymph node involvement in order to refine the identification of patients at high-risk.

To this end we conducted this retrospective study with the aim to identify prognostic histopathological and immunohistochemical markers using tissue microarrays (TMAs) from patients with AEG/S stage UICC I-N0 and correlated these data with overall survival and tumor related survival. Refined biomarkers in this subgroup might not only be predictive for survival, but might guide treatment to improve survival in high-risk patients by possibly intensifying treatment by adjuvant chemotherapy or chemoradiotherapy.

## Material and Methods

### Patients

Clinical data from patients with AEG/S stage pT1 and pT2 pN0M0, treated solely by surgery between September 1993 and May 2010 at *Charité—University Medicine Berlin*, were collected retrospectively.

The data including patient characteristics and follow-up information were retrieved from the patient management software (SAP®) and the regional population-based cancer registry (*Gemeinsames Krebsregister*). This study was approved by the Institutional Ethical Review Board of the *Charité* (EA4/115/10).

All procedures followed were in accordance with the ethical standards of the responsible committee on human experimentation (institutional and national) and with the Helsinki Declaration of 1964 and later versions. No patient consent was necessary since this was a retrospective study. Patients’ records were collected and anonymized, de-identified before the analysis.

Patients without recurrent disease were followed-up for a minimum period of 24 months. Overall survival, used as a measure of prognosis, was defined as time from diagnosis to death or last follow-up. Tumor related survival was defined as time from diagnosis to tumor related death or last follow-up.

### Tissue Samples

Tissue samples were collected from the pathological archive of the *Charité—University Medicine Berlin*. From 129 surgically treated patients 80 paraffin embedded tumor samples were available. A specialist in gastrointestinal pathology (H.B.) reanalyzed and reconfirmed the primary postoperative histological diagnosis and tumor stage and classified the histological architecture of gastric carcinoma using Lauren’s, Ming and WHO classification [34]. According to the WHO classification for colon and rectum carcinoma we added the subtype of medullary tumors to our analysis [34–36]. We defined the poorly differentiated medullary cancer (PMC) subtype in congruence with Adachi et al. as a tumor characterized by sheets of monomorphic malignant cells showing solid growth and absence of glandular growth [35]. Tumor cells show only little pleomorphism, have round to oval, large nuclei and a high nuclear to cytoplasmic ratio, morphologically resembling medullary type colorectal cancer. The PMCs did not show a deficiency of the mismatch repair system, as tested by immunohistochemistry.

Additional data concerning tumor size, depth of invasion, and tumor invasion of veins or lymphatic vessels were extracted from the *Charité—University Medicine* patient management software.

### TMA and Immunohistochemistry

Tissue samples were screened in a HE-stained section for representative areas of solid tumors. Two 1 mm-diameter tissue cores were punched out from each of the 80 available cases and were transferred to a recipient paraffin block. Tissue from a tonsil was used as positive staining control and was also transferred to the same paraffin block. After re-melting, sections (4 μm-thick) were consecutively cut from each tissue microarray block. HE staining was performed on TMA sections for confirmation of tumor and non-tumor tissue.

Immunohistochemical analysis was performed on TMA sections using monoclonal antibodies. For pre-treatment a target retrieval solution (TRS) (HIF1A) or citrate buffer (pH = 6.0, 0.1M) was used. Antibody binding was visualized using an APAAP-Kit (K5000 Dako®) for E-cadherin (1:50, clone 36B5, Novocastra®), Ki67 (1:200, Clone MIB-1, Dako®), BCL2 (1:25, clone 124, Dako®), HIF1A (1:100, Clone Halpha111a, BioZol®); K5005 Dako® for CDX2 (1:600, clone CDX2-88, Biogenex®), CD3 (1:100, clone LN10, Novocastra®) TP53 (1:100, clone DO-7, Dako®), TP21 (1:10, clone DCS-60.2, Thermo Scientific®), BAX (1:500, Clone A3533, Dako®, Cyclin E (1:100, clone HE12, Invitrogen®), Survivin (1:200, clone 12C4, Dako®), SSH (1:500, clone EP1190Y, Dako®) and a biotin labeled anti-goat IgG Antibody (Dako E0466) for TROP2 (1:100, clone P09758, R&D Systems®). For the mismatch-repair proteins immunohistochemistry was performed on an automated staining system (BenchMark Ultra, Roche Ventana, Germany) using prediluted antibodies (all Roche Ventana) MLH1 (clone M1), PMS2 (clone EPR3947), MSH2 (clone G219-1129), and MSH6 (clone 44) following the manufacturer’s instructions.

Membrane, cytoplasmatic and nuclear stained cells with E-Cadherin, BCL2, HIF1A, BAX, SSH and solely nuclear stained cells with Cyclin E, TP21, CDX2 were scored according to the intensity of staining (0, none to weak; 1 = weak; 2 = moderate; 3 = strong) and the percentage of tumor cells stained (0 = 0% positive; 1 = 1–25% positive, 2 = 26–50% positive; 3 = 51–75% positive and 4, > 75% positive). Tumor samples with a score higher than 3 were evaluated as positive cases.

Concerning Ki-67, tumors with a proliferation rate higher than 20% were regarded as highly proliferative tumors. For Survivin, all tumors with more than 5 percent positively stained cell nuclei were scored as Survivin positive samples [37]. A high number (75–100%) of TP53 positive stained cells with a strong nuclear staining intensity or tumors with no TP53 positive cells was used as a surrogate parameter for TP53 mutated samples [38,39].

Mismatch repair deficiency was scored, when a tumor showed specific loss of nuclear staining for at least one of the markers (MLH, PMS2, MSH2, MSH6)

The count of CD3 positive cells was performed in 4 representative regions of the tumor. The average of counted cells was normalized in 0.1mm^2^ of tumor area.

Each core was blindly scored by two independent evaluators (C.T. and S. D.) and reviewed by one pathologist (H.B.) and one biologist highly experienced in pathohistology (M.H.) and reconciled after intensive discussion.

### Statistics

Quantitative values are expressed as mean± standard deviation, median, and range, and categorical values with absolute and relative frequencies (count and percent). Overall survival was evaluated in months from time of diagnosis until death or until the most recent follow-up using the Kaplan–Meier plots. Differences in the survival curves were evaluated by the log-rank test for significance. The X^2^-test was used for comparison of frequencies. A p-value < 0.05 was considered as statistically significant. IBM SPSS Version 21 (Ehningen, Germany) was used for statistical analysis.

Reporting recommendations for tumor marker prognostic studies (REMARK) were applied for this study whenever applicable [40].

## Results

### Clinical Characteristics of Patients

Data of 129 patients (female = 48, age 29–90 years, median age 62.9 years) were retrieved for this study (see Table 1). The cohort consisted of 96 patients with gastric cancer (2 pts. upper third, 26 middle third, 55 lower third, 8 couplet stomach and 5 with no specified position in the stomach) and 33 patients with carcinoma of the gastroesophageal junction. A D2 lymph node dissection was performed in 97.6% (126 patients) of AEG and stomach resections (two patients had only a D1 dissection and in one patient lymph node dissection status was not reported.)

**Table 1:** Patient characteristics collected from the patient management software and the regional population-based cancer registry (n = 129).

	Pts. (%)	Tumor related Survival (%)			Overall survival (%)		
		5-year	10-year	Log Rank	5-year	10-year	Log Rank
**Sex**							
Female	48 (37.2%)	95.8	93.0	0.713	91.6	74.8	0.569
Male	81 (62.8%)	94.7	91.0		79.0	69.7	
**Tumor size**							
T1	88 (68.2%)	95.4	95.4	0.153	86.7	77.5	0.225
T2	41 (31.8%)	94.8	85.6		79.3	61.6	
**Tumor Location**							
AEG	33 (25.6%)	90.5	85.2	0.519	81.8	71.1	0.266
S 1/3	2 (1.6%)	100	100		50.0	50.0	
S 2/3	26 (20.2%)	100	94.4		92.3	87.2	
S 3/3	55 (42.6%)	94.3	92.6		87.1	75.5	
S total	8 (6.2%)	100	100		100	100	
Unspecified	5 (3.9%)	-	-	-	-	-	-
**Lymphatic Vessel Invasion**							
L0	66 (51.2%)	93.5	88.3	0.369	78.4	65.1	0.971
L1	14 (10.9%)	80.0	80.0		75.0	60.0	
Unspecified	49 (38.0%)	-	-	-	-	-	-
**Vein Invasion**							
V0	63(48.8%)	96.7	91.3	0.005	85.9	71.3	0.165
V1	8 (6.2%)	60.0	60.0		42.9	42.9	
Unspecified	58(45.0%)	-	-	-	-	-	-
**Grading**							
G1	12 (9.3%)	100	100	0.495	100.0	83.3	0.937
G2	42 (32.6%)	92.7	86.5		82.1	65.4	
G3	71 (55%)	95.5	93.5		80.9	70.9	
G4	1 (0.8%)	100	100		0.0	0.0	
Unspecified	3 (2.3%)	-	-	-	-	-	-
**Resection**							
R0	98 (76.0%)	94.6	89.8	**<0.001**	82.2	68.0	0.001
R1	1 (0.8%)	0.0	0.0		0.0	0.0	
Unspecified	30 (23.3%)	-	-	-	-	-	-
**Lymph node Resection**							
D2	126 (97.6%)	94.7	91.7	0.758	88.7	78.3	0.473
D1	2 (1.6%)	100	100		50.0	50.0	
Unspecified	1 (0.8%)	-	-	-	-	-	-
**Lauren Classification**							
Intestinal	46 (35.7%)	93.3	90.5	0.533	82.0	64.0	0.721
Diffuse	36 (27.9%)	91.4	87.3		81.5	72.4	
Mixed	11 (8.5%)	100	100		75.0	62.5	
Unspecified	36 (27.9%)	-	-	-	-	-	-
**Relapse**							
Yes	9 (7%)	-	-	-	33.3	0.0	<0.0001
No	120 (93%)	-	-	-	89.2	79.7	
**Total**							
	129 (100%)	95.1	91.8	-	83.9	71.4	-

The mean follow-up was 129.1 months and 21 patients (16.3%) died during the follow-up period, but only nine patients (7%) died from recurrence of AEG/S.

**Fig 1:**
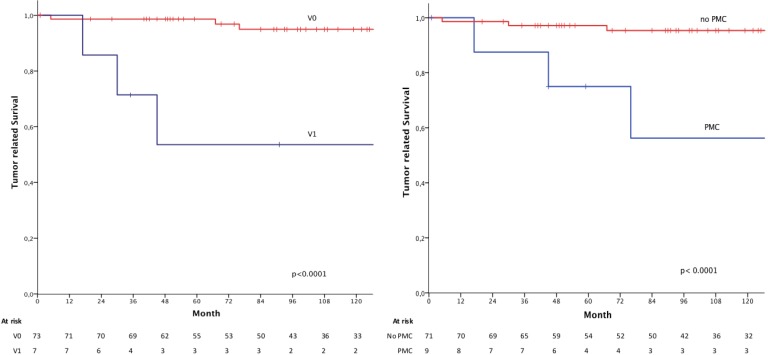
Kaplan–Meier estimates of tumor related survival measured from the date of first admission stratified by vein invasion status V0 (red) /V1(blue) (left panel) and poorly differentiated medullary cancer (PMC) (blue) vs. other histopathological subtypes (no-PMC) (red) (right panel).

The 5-year overall survival of our population was 83.9%, the 5-year tumor related survival was 95.1% (Fig 1). A relapse of AEG/S occurred in nine patients whereas 120 patients remained relapse-free in the observation period.

A stage pT1N0M0 was assigned to 68.2% of patients (88/129), and 31.8% of patients (41/129) were pT2N0M0. In 80 cases the information of lymphatic vessel invasion was described and 14 (10.9%) were staged as L1 and 66 (51.2%) as L0. In 71 cases the vein invasion level was documented. Eight patients (6.2%) were staged as V1 and 63 (48.8%) as V0 (Table 1).

### Histopathological Characteristics

From 129 patients with clinical data, 80 formalin-fixed paraffin embedded tissue samples were available and reassessed (Table 2).

**Table 2:** Histopathological characteristics of the 80 tumor samples reevaluated from available formalin-fixed paraffin-embedded tissue samples. PMC: Poorly differentiated medullary cancer.

	Pts. (%)	Tumor related Survival (%)			Overall survival (%)		
		5-year	10-year	Log Rank	5-year	10-year	Log Rank
**Tumor size**							
T1a	31 (38.8%)	93.4	93.4	0.952	88.9	88.9	0.179
T1b	26 (32.5%)	100.0	90.5		94.4	88.9	
T2	23 (28.8%)	90.6	90.6		73.7	67.5	
**Vein Invasion**							
V0	73 (91.3%)	98.6	95.0	**<0.001**	92.1	85.9	**0.001**
V1	7 (8.8%)	53.6	53.6		42.9	42.9	
**Lymphatic Vessel Invasion**							
L0	66 (82.5%)	96.8	92.6	0.294	89.3	82.3	0.672
L1	14 (17.5%)	85.1	85.1		78.6	78.6	
**Grading**							
G1	7 (8.8%)	100	100	0.774	100	100	0.589
G2	27 (33.8%)	96.2	91.6		88.8	83.1	
G3	46 (57.5%)	93.2	90.5		84.9	78.6	
**Lauren Classification**							
Intestinal	56 (70.0%)	92.6	88.0	0.280	86.2	80.7	0.282
Diffuse	13 (16.3%)	100	100		81.8	71.6	
Mixed	11 (13.8%)	100	100		100	100	
**WHO Classification (modified)**							
Tubular	40(50.0%)	97.5	97.6	**0.004**	91.9	84.0	0.420
Papillary	3 (3.8%)	66.7	66.7		66.7	66.7	
Mucinous	2 (2.5%)	100	100		50.0	-	
PMC	9 (11.3%)	75.0	56.3		80.0	60.0	
Pyloric gland type	4 (5.0%)	100	66.7		75.0	75.0	
Poorly cohesive (signet ring)	15 (18.8%)	100	100		85.7	77.9	
Mixed	7 (8.8%)	100	100		60	60	
**Ming Classification**							
Expansive	38 (47.5%)	91.7	88.3	0.175	86.3	82.7	0.903
Infiltrative	37 (46.3%)	97.4	97.4		88.4	80.8	
Not possible	5 (6.3%)	-	-	-	-	-	-
**Total**							
	**80 (100%)**	**94.8**	**91.4**	**-**	**83.9**	**71.4**	**-**

The reevaluation by a specialist in GI pathology (H.B.) resulted in 31 (38.8%) tumors staged as T1a, 26 (32.5%) as T1b and 23 (28.8%) as T2. Seven (8.8%) tumors showed vein invasion and 14 (17.5%) a lymphatic vessel invasion, while seven (8.8%) patients had G1, 27 (33.8%) G2, and 46 (57.5%) G3 tumors.

**Fig 2:**
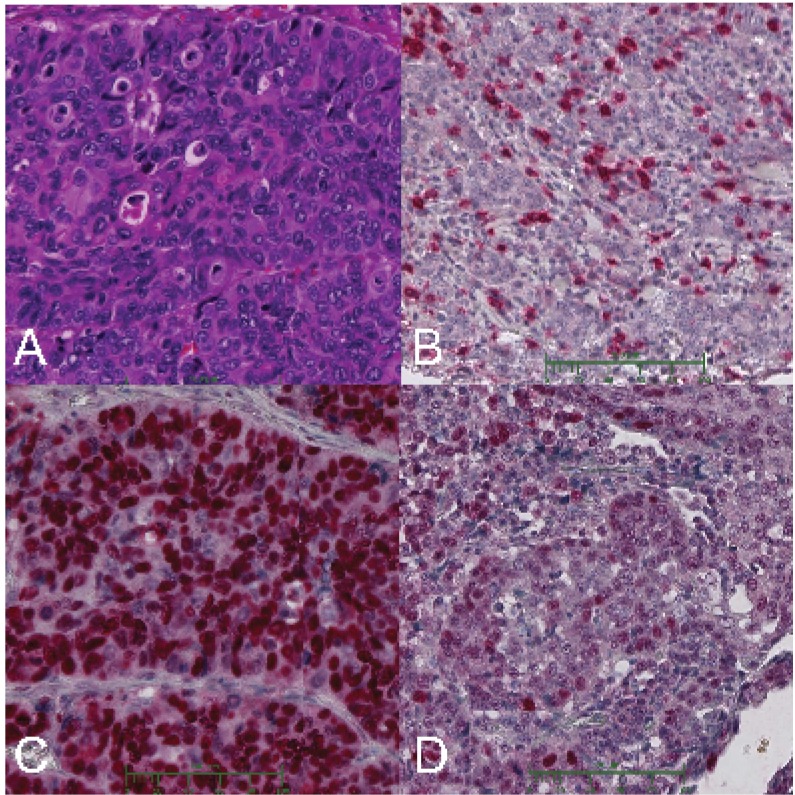
Immunhistochemical and H&E staining of the TMA of gastric cancer specimens (200x). Poorly differentiated medullary cancer by H&E staining (A) and CD3 staining (B). TP53 mutated (C) and wildtype (D) tumor sample.

The histopathological classification according to Lauren yielded 56 (70.0%) intestinal, 13 (16.3%) diffuse and 11 (13.8%) mixed type tumors. The WHO Classification resulted in: 40 (50.0%) tubular histology, 9 (11.3%) medullary, 3 (3.8%) papillary, 4 (5.0%) pyloric gland type, 2 (2.5%) mucinous, 15 (18.8%) signet ring and 7 (8.8%) mixed type tumors (medullar subtype see Fig 2A and 2B). Using the Ming classification, tumors were in 38 cases (47.5%) of the expansive and in 37 cases (46.3%) of the infiltrative type. In five cases (6.3%) a definite classification was not possible (Table 2).

The correlation of histopathological characteristics with tumor related survival or overall survival showed significant differences between the overall survival and tumor related survival for the V0 and V1 subpopulation (5-year tumor related survival V0 96.7%, V1 60.0% p<0.001; 5-year overall survival V0 85.9%, V1 42.9% p = 0.001) (Table 2; Fig 1). Furthermore, we could identify significant differences between poorly differentiated medullary cancer (PMC) subtypes and non-PMC tumors (PMC vs. non-PMC: 5-year-overall survival 57.1% vs 87.8%; p = 0.007, 5-year tumor related survival 75.0% vs 97.1%; p<0.001) (Fig 1). Tumor grading, Lauren or Ming classification were no discriminating factors for survival (Table 2).

A multivariate cox regression model was not reasonable as the number of our cases, especially of relapsed cases, did not meet the critical number proposed by Peducci et al [41].

### Immunohistochemistry

Although all cases were present in duplicate, between 12.5 to 20% (10–20) of the cases were not usable for analysis in the TMA. Reasons for exclusion were cores with less than 10% tumor tissue.

In the analysis of the immunhistochemical markers BAX, BCL-2, CDX2, Cyclin E, E-Cadherin, Ki-67, TP53, TP21, SHH, Survivin, HIF1A, and TROP2 we could not find a significant correlation with marker expression and prognosis (see Table 3.).

**Table 3:** Immunohistochemical biomarker analysis: All immunohistochemical markers except Ki-67, TP53, and Survivin were analyzed using the semiquantitative scoring system described in the method section.

	Pts. (%)	Tumor related survival (%)			Overall survival (%)		
		5-year	10-year	Log Rank	5-year	10-year	Log Rank
**BAX**							
Negative	15 (18.8%)	100	100	0.179	85.1	76.6	0.624
Positive	53 (66.3%)	92.0	86.9		83.6	72.8	
Excluded	12 (15.0%)	-	-	-	-	-	-
**BCL-2**							
Negative	53 (66.3%)	94.1	89.0	0.907	87.2	76.2	0.674
Positive	12 (15.0%)	91.7	91.7		83.3	83.3	
n.a.	15 (18.8%)	-	-	-	-	-	-
**CDX2**							
Negative	28 (35.0%)	89.1	84.9	0.506	84.1	75.2	0.742
Positive	37 (46.3%)	97.1	93.0		82.4	70.6	
n.a.	14 (17.5%)	-	-	-	-	-	-
**Cyclin E**							
Negative	34 (42.5%)	90.0	82.6	0.170	87.0	73.7	0.973
Positive	25 (31.3%)	95.7	95.7		83.3	77.8	
n.a.	21 (26.3%)	-	-	-	-	-	-
**E-Cadherin**							
Negative	13 (16.3%)	100	100	0.181	87.5	87.5	0.093
Positive	52 (65.0%)	91.7	86.2		78.8	67.9	
n.a.	15 (18.8%)	-	-	-	-	-	-
**HIF1A**							
Negative	62 (77.5%)	93.3	88.9	0.416	86.4	67.2	0.751
Positive	7 (8.8%)	100	100		85.7	68.6	
n.a.	11 (13.8%)	-	-	-	-	-	-
Ki67*							
Low proliferative	22 (27.5%)	100	100	0.075	100	100	0.012
High proliferative	42 (52.5%)	90.2	84.0		79.9	67.8	
n.a.	16 (20.0%)	-	-	-	-	-	-
**TP21**							
Negative	23 (28.8%)	95.7	95.7	0.275	85.9	79.7	0.453
Positive	37 (46.3%)	91.4	84.7		85.2	70.1	
n.a.	20 (25.0%)	-	-	-	-	-	-
TP53**							
Wt pattern	43 (53.8%)	97.7	94.7	0.057	92.5	80.2	**0.044**
Mutation pattern	23 (28.8%)	85.2	78.7		66.3	60.3	
n.a.	14 (17.5%)	-	-	-	-	-	-
**SHH**							
Negative	14 (17.5%)	100	100	0.220	90.9	77.9	0.493
Positive	56 (70.0%)	92.4	87.7		82.5	73.1	
n.a.	10 (12.5%)	-	-	-	-	-	-
Survivin***							
Negative	63 (78.8%)	93.4	89.3	0.560	86.2	77.2	0.390
Positive	3 (3.8%)	100	100		100	100	
n.a.	14 (17.5%)	-	-	-	-	-	-
**TROP2**							
Negative	5 (6.3%)	100.0	100.0	0.466	100.0	50.0	0.837
Positive	63 (78.8%)	93.3	89.0		82.8	74.8	
n.a.	12 (15.0%)	-	-	-	-	-	-
**Mismatch repair system**							
Proficient	53 (66.3%)	92.1	87.3	0.461	82.0	72.0	0.842
Deficient	5 (6.3%)	100	100		75.0	75.0	
n.a.	22 (27.5%)	-	-	-	-	-	-
**Total**							
	**80 (100%)**	**94.8**	**91.4**	-	**83.9**	**71.4**	-

*Ki-67: Samples > 20% positive cells were evaluated as high proliferative tumors.

** TP53 Samples with 75–100% stained cells and strong nuclear staining intensity were scored as TP53 mutated.

***Survivin: tumors with >5% positive stained nuclei were scored as positive.

n.a.: not assessable

In a more detailed analysis for positive and negative markers we detected a lower 5-year overall survival for Ki-67 high proliferative tumors (high: 79.9% vs. low: 100% p = 0.012) and for tumors with TP53 surrogate mutation pattern (mut 66.3% vs. wt 92.5%; p = 0.044). These markers showed a trend in the 5-year-tumor related survival analysis (Ki-67 high 90.2% vs. low 100% and TP53 mut: 85.2% vs wt 95.7%), but did not reach level of significance (Ki-67 p = 0.075 and TP53 p = 0.057).

The analysis of mismatch repair system deficiency and proficiency did not show any significant differences for overall survival or tumor related survival (5-year tumor related survival dMMR 100% vs. pMMR 92.1%, p = 0.461; 5-year overall survival dMMR 75.0% vs pMMR 82.0%, p = 0.842)

With regard to the above identified histomorphological risk factors (poorly differentiated medullary cancer subtype, venous invasion) we found in PMC significantly more T2 tumors (p = 0.034), more undifferentiated tumors (p = 0.024) and more p53 mutated tumors (p = 0.045). None of the PMC showed a deficient MMR.

For detailed classification of the PMC we counted the CD3 positive lymphocyte per 0.1mm^2^ tumor area. We detected less CD3 positive cells in the PMC compared to non-PMC tumors (120.5 per 0.1mm^2^ vs 212.3 per 0.1mm^2^). The difference did not reach level of significance (p = 0.1).

T2-stage was associated with positive venous invasion (p = 0.033) and p53 mutated subtype (p = 0.003). Furthermore, we found a high correlation between V1 and L1 status (p>0.0001).

## Discussion

The aim of this retrospective study was to identify prognostic histopathological and immunohistological markers for patients with AEG/S stage UICC I on top of the known main risk factor lymph node metastasis to identify potential high risk subgroups.

Detailed clinical data were retrieved for 129 patients and histopathological data were assessable in 80 cases in this study. This Caucasian UICCI/N0 patient cohort with FFPE samples is one of the largest of gastric cancer in Europe as the incidence is substantial lower and tumor stages are more advanced in comparison to Eastern Asia [42,43].

From 129 patients followed over 10 years we found 9 patients with tumor related death. This high 5-year tumor-related overall survival of 95.1% proves the effectivity of surgical treatment in this early stage disease without lymph node involvement. Similar favorable data are well known from Asian patients (97.8% 5-year tumor-related overall survival) [44]. Similar results in Caucasians were reported in a single study by Borie et al. with a 92% 5-year tumor-related overall survival [8]. The large German gastric cancer trial showed in about 250 patients recruited from 1986–1989 a 5-year overall survival depending on the number of resected lymph nodes from 81%-85% in T1N0 stages to 67% in T2N0 stages [6]. These differences in survival are probably attributable to increased surgical quality in the last decades and the decreased postoperative 30 day mortality rate (mean 30 day mortality rate of 0.8% in 18 trials between 1998–2014 [45] compared to the German gastric cancer trial with 5.2% [3]).

For the histopathological markers in our study, venous infiltration was a significant predictor for tumor related death in our patient group (5 year-tumor related survival V0 96.7%, V1 60.0% p = 0.005). While these data are in accordance with Asian studies [10,11], we found a higher number of patients with poorly differentiated medullary cancer subtype, namely 11.3% (9/80) whereas the Asian data reported about 3.0% of PMC histology, defined as low differentiated lymphoepithelioma-like tumor type with a small amount of stromal tissue [35,36]. Although definition of medullary subtype is still a point of discussion, our defining criteria are easily reproducible. Similar to Asian patients, PMC subtype was associated with relapse and reduced prognosis in our patient population [35,36]. Although PMC phenotype (see Fig 2A and 2B) is not part of the classification systems currently in use (WHO, Ming, Lauren), it seems to represent an atypical form of gastric cancer which should be studied further, considered as a separate entity, and included in histopathological classification systems [46].

According to our data, all other parameters analyzed, like Lauren and Ming classification, tumor size, grading, lymph vessel invasion, and signet ring cell type, did not show a prognostic relevance. As other authors found grading, lymph vessel invasion and tumor size as prognostic in early tumor stages mainly with lymph node involvement [10–14], employing a more detailed analysis (N0 versus N+) of the tumor, might explain these differences.

The conclusion from our immunohistochemical data for prognostic biomarkers is somewhat limited by the rather small number of cases, solely 58–70 from 129 included patients could be analyzed, and the number of patients with tumor related death among this group amounts to six patients (see Table 3.). A reduced overall survival for TP53 surrogate mutation pattern and for high proliferative (Ki-67>20%) tumors could be demonstrated. The identified histopathological risk factors vein invasion and PMC subtype were positively correlated with a TP53 mutation pattern. TP53 and Ki-67 did not reach the level of significance in correlation with disease relapse in contrast to correlation with death by any cause.

Yet, the results for TP53-mutating pattern and high proliferating tumors are in concordance with other studies. Over all stages of gastric cancer Ki-67 is associated with metastasis and disease progression in gastric cancer and is especially associated with lymph node involvement in early diseases [47,48].

Also TP53 mutation is known to be associated with reduced survival [15,49,50] and the mutation of TP53 plays a crucial role in the progression from intestinal metaplasia to gastric cancer [49,51]. Despite missing significance (p = 0.057) for this biomarker, the immunohistochemical TP53 surrogate mutation pattern seems to be a suitable molecular biomarker for early gastric cancer.

This retrospective study in a non-selected group of patients with early adenocarcinoma of the esophago-gastric junction and stomach corroborates older findings of a favorable prognosis in cases without lymph node involvement. We could confirm the negative prognostic role of vein invasion in our population despite the small number of patients with relapse. Furthermore, we could identify the subtype of poorly differentiated medullary cancer as a risk factor for disease recurrence in a Caucasian population.

The prognostic role of these findings has to be investigated in more detail in further adequately powered prospective studies.

## Supporting Information

S1 Table Clinical data and IHC scoring value for each marker. (XLSX)
